# A taxonomy of quality assessment methods for volunteered and crowdsourced geographic information

**DOI:** 10.1111/tgis.12329

**Published:** 2018-04-19

**Authors:** Lívia Castro Degrossi, João Porto de Albuquerque, Roberto dos Santos Rocha, Alexander Zipf

**Affiliations:** ^1^ Institute of Mathematics and Computer Science University of São Paulo São Carlos Brazil; ^2^ Centre for Interdisciplinary Methodologies University of Warwick Coventry United Kingdom; ^3^ Institute of Geography Heidelberg University Heidelberg Germany

## Abstract

The growing use of crowdsourced geographic information (CGI) has prompted the employment of several methods for assessing information quality, which are aimed at addressing concerns on the lack of quality of the information provided by non‐experts. In this work, we propose a taxonomy of methods for assessing the quality of CGI when no reference data are available, which is likely to be the most common situation in practice. Our taxonomy includes 11 quality assessment methods that were identified by means of a systematic literature review. These methods are described in detail, including their main characteristics and limitations. This taxonomy not only provides a systematic and comprehensive account of the existing set of methods for CGI quality assessment, but also enables researchers working on the quality of CGI in various sources (e.g., social media, crowd sensing, collaborative mapping) to learn from each other, thus opening up avenues for future work that combines and extends existing methods into new application areas and domains.

## 1 INTRODUCTION

The use of crowdsourced geographic information (CGI) has grown in the past few years, owing to a number of key features (e.g., it is free, up‐to‐date, and provided by several volunteers). CGI is being used as an umbrella term that encompasses both “active/conscious” and “passive/unconscious” georeferenced information generated by non‐experts (See et al., 2016). This term has been used as a broader replacement for volunteered geographic information (VGI) (Goodchild, 2007), since the term “volunteered” does not seem appropriate to refer to information that is collected without the will or conscious knowledge of the provider (Harvey, 2013).

When making use of CGI, ensuring the quality of the information is a challenging question. The information that is supplied by non‐experts mostly does not have to comply with any quality standards, and there is no centralized control over the creation process. Quality assessment thus becomes an important step to understand if the information is fit‐for‐purpose with regard to the way it will be used (Ballatore & Zipf, 2015).

The quality of CGI has become a very popular topic amongst academics and researchers (Antoniou & Skopeliti, 2015). Several researchers have investigated approaches to assess the quality of CGI, so that there is currently a large number of methods to accomplish this task (e.g., Foody et al., 2013; Girres & Touya, 2010; Senaratne, Bröring, & Schreck, 2013). These methods differ with regard to the type of information evaluated, and reference data types, among other factors. Owing to the large number of existing methods, selecting the most appropriate one for a particular purpose is not a trivial task.

In an attempt to summarize the existing methods in the literature, some previous studies have reviewed and categorized them (Bordogna, Carrara, Criscuolo, Pepe, & Rampini, 2016; Mirbabaie, Stieglitz, & Volkeri, 2016; Senaratne, Mobasheri, Ali, Capineri, & Haklay, 2017; Wiggins, Newman, Stevenson, & Crowston, 2011). However, these previous studies do not differentiate clearly between assessment methods that require a reference dataset and methods that can be used when no comparable data is available. The latter is probably the most frequent situation in practice, since the use of CGI is often motivated by a lack of availability (or currency) of authoritative data sources.

Against this backdrop, this article is motivated by the following research question: *What types of methods can be employed to assess the quality of CGI in the absence of authoritative data?* We address this question by designing a taxonomy of the existing methods in the literature. The purpose of this taxonomy is to form a basis for the researchers and designers of collaborative platforms based on CGI, so that they can select the best method for their purposes, by discussing the idiosyncrasies of each method. Additionally, the taxonomy can be used to systematically summarize research results in the literature and determine where further investigation is still needed.

The remainder of this article is structured as follows. Section 2 presents background concepts on the quality of CGI. In Section 3, there is an overview of related works. In Section 4, the methodology employed for the development of the taxonomy is described. Following this, the proposed taxonomy is described in detail in Section 5. In Section 6, we discuss our findings and the limitations of our taxonomy, and make suggestions for future research. Finally, in Section 7, we summarize our conclusions.

## 2 QUALITY OF CROWDSOURCED GEOGRAPHIC INFORMATION

The quality of CGI largely depends on different factors, such as the characteristics of the volunteer, the type of information, and the way in which the information is produced (Bordogna et al., 2016). CGI is provided by a wide range of sources (i.e., volunteers), who have different levels of expertise and come from different backgrounds, which can be classified into three types of collaborative activities (Albuquerque, Herfort, Eckle, & Zipf, 2016), described as follows.

###### Social media

The first type of collaborative activity comprises volunteers sharing geographic information on social media, which are Internet‐based applications built on the ideological and technological foundations of Web 2.0 (Kaplan & Haenlein, 2010). Volunteers use social media to share their experience and/or opinion in “feeds” or “messages,” which may contain a geographic reference and thus be used as a source of “ambient geographic information” (Stefanidis, Crooks, & Radzikowski, 2013).

###### Crowd sensing

The second type of collaborative activity comprises the use of collaborative technologies for gathering “in situ” observations by means of specific platforms. The term “people as sensors” (Resch, 2013) and related forms of “citizen science” are also associated with the activities we are considering here. They mostly rely on dedicated software platforms and their purpose is to collect specific and structured information observed “on the ground.”

###### Collaborative mapping

The third type of collaborative activity entails generating a particular type of digital data (i.e., data about “geographic features,” which we understand as characteristics of the geographic space). This type of activity requires volunteers to produce a very specific type of georeferenced data, for instance geographic data about points of interest, streets, roads, buildings, land use, etc. In our previous work, we proposed classifying the collaborative mapping tasks performed into three types of analytical tasks (Albuquerque, Herfort, & Eckle, 2016). In classification tasks, volunteers analyze an existing piece of geographic information and classify it into a category that better represents it. For instance, this might involve volunteers interpreting satellite imagery to classify land cover (Salk, Sturn, See, Fritz, & Perger, 2016). In digitization tasks, volunteers create geographic data (including a geometry and a location) of a real‐world geographic object, for instance by digitizing building footprints (Mooney & Minghini, 2017). Finally, in conflation tasks, volunteers analyze and interpret geographic information from multiple sources, conflating them to find matching features/objects and thus produce new geographic information (e.g., detecting changes in geographic objects) (Anhorn, Herfort, & Albuquerque, 2016).

These types of activity are referred to in various ways in the literature, and it is not our intention here to be exhaustive (for a discussion on this, see See et al., 2016). In giving this summary of the types of collaborative activity required for the production of crowdsourced geographic information, we would like to set up a context to discuss issues related to the quality of the information. This summary shows that the different types of collaborative activity result in geographic data with varying degrees of accuracy, structure, and format standardization. For instance, while geotagged social media messages may have heterogeneous formats (e.g., text‐, image‐, and map‐based data) and varied structure, some projects employ a CGI standardized process for gathering CGI (e.g., Salk et al., 2016). However, even with more standardized data formats and procedures, the extent to which volunteers will adhere to them is uncertain. As a result, CGI is often suspected of having a heterogeneous quality and uncertain credibility (Flanagin & Metzger, 2008), which might affect the usability of the crowdsourced information (Bishr & Kuhn, 2013). The quality of CGI also depends on how the information is used, since the quality of the information is determined by its “fitness for use” within the context in which it is applied (Bordogna et al., 2016).

In the literature, the quality of CGI is often measured by making reference to the “quality elements” that are traditionally used to assess the quality of geographic information (International Organization for Standardization, 2013). These quality elements include completeness, positional accuracy, and thematic accuracy, among others. Although these elements can be applied to measure the quality of CGI, this type of information has particular features which make assessing its quality different from traditional geographic data (Mohammadi & Malek, 2015). Hence, researchers have added new elements to assist in assessing the quality of CGI (e.g., trust), or made new definitions for existing quality elements (Fan, Zipf, Fu, & Neis, 2014; Girres & Touya, 2010).

These elements can be measured by means of different methods. Particularly, Goodchild and Li (2012) proposed three approaches to assess the quality of CGI when authoritative data is not available. The *crowdsourcing approach* relies on the ability of a group of individuals (peers) to validate and correct erroneous information that another individual might provide. In this sense, the term crowdsourcing (might) have two interpretations that are relevant to quality assurance of CGI. In the first interpretation, quality might be assured on the basis of the number of independent but consistent observations (i.e., an item of information can be strengthened by additional information, reporting the same event from the same or nearby landmarks/points). For example, CGI reporting a flood event can be strengthened by additional information from the same point or a point in the surrounding area. In the second interpretation, quality can be assured in terms of the ability of the crowd to converge on the truth. In OpenStreetMap, for instance, if erroneous geographic information is provided, individuals (peers) are expected to edit and correct it (Mooney & Minghini, 2017).

The *social approach* could also be called hierarchical approach since it relies on a hierarchy of individuals who act as moderators or gatekeepers of crowdsourcing platforms. Thus, quality is assured by a group of people who maintain platform integrity, prevent vandalism and infringement of copyright, and avoid the use of abusive content. In the Flood Citizen Observatory (Degrossi, Albuquerque, Fava, & Mendiondo, 2014), for instance, the platform administrator acts as a gatekeeper by assessing the veracity of CGI and classifying it as checked or unchecked. Finally, the *geographic approach* involves comparing an item of geographic information with the body of geographic knowledge. Thus, this approach adheres to rules such as the First Law of Geography, where “everything is related to everything else, but near things are more related than distant things” (Tobler, 1970). Geographic information should, for instance, be consistent with what is known about the location and surrounding area. In other words, it should be related to the space in which the knowledge has been provided. For instance, Albuquerque, Herfort, Brenning, and Zipf (2015) showed that when the overall number of flood‐related tweets are compared, there is a tendency for “relevant” on‐topic tweets to be closer to flood‐affected catchments.

Depending on which reference dataset is used, quality assessment methods can be classified as either extrinsic or intrinsic. Extrinsic methods use external knowledge to measure the quality of CGI. Although authoritative data are commonly used as external knowledge, their use can be constrained by financial costs, licensing restrictions (Mooney, Corcoran, & Winstanley, 2010), and currency (Goodchild & Li, 2012). On the other hand, intrinsic methods do not rely on external knowledge for assessing the quality of CGI. These methods may, for instance, analyze historical metadata as a means of inferring the inherent quality of the data. Thus, it is possible to evaluate the quality of CGI regardless of whether a reference dataset is available or not. However, in most cases, intrinsic methods do not allow absolute statements to be made about CGI quality. Thus, they can only be used for making rough estimates of the possible data quality (Barron, Neis, & Zipf, 2014). Barron et al. (2014), for instance, proposed new intrinsic methods and indicators for assessing the quality of OpenStreetMap data.

Furthermore, quality assessment methods can be employed in the light of two temporalities: (a) *ex ante;* and (b) *ex post* (Bordogna et al., 2016). These differ with regard to the time when the assessment is carried out compared with the creation time of CGI. The *ex‐ante* strategy is employed before a CGI is created and seeks to avoid the creation of low‐quality CGI (Bordogna et al., 2016). As well as offering mechanisms for controlling data creation, these methods also provide volunteers with resources for guiding the way information is produced. In contrast, the *ex‐post* strategy is employed after a CGI item has been created. This strategy aims at removing and improving CGI quality. This involves first checking the quality of CGI and, later, filtering it.

## 3 RELATED WORKS

**Figure 1:**
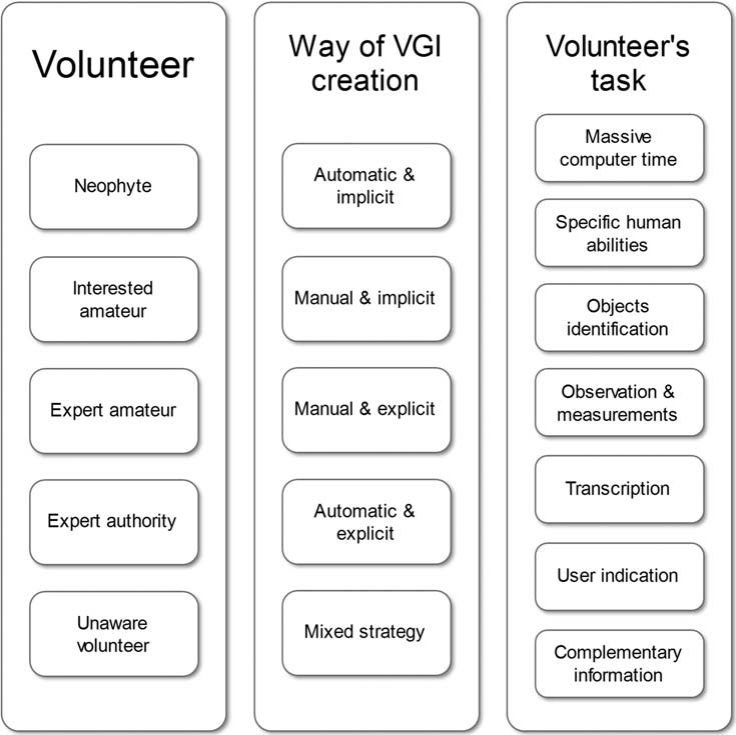
Classification schema of quality assessment methods *Source*: Adapted from Bordogna et al. (2016)

Several critical literature reviews (or surveys) involving the categorization of quality assessment methods have been conducted to provide an overview of this area (e.g., Bordogna et al., 2016; Mirbabaie et al., 2016; Senaratne et al., 2017; Wiggins et al., 2011). An analysis of the quality assessment methods was carried out by Wiggins et al. (2011), where the authors analyzed the data validation policy and quality assessment in citizen science projects (i.e., crowd sensing). They found that the most common type of data validation is based on expert reviews conducted by trusted individuals or moderators. Bordogna et al. (2016) also analyzed CGI in citizen science projects. They initially reviewed and categorized CGI projects, by analyzing the way they deal with CGI quality. This work also provided a classification scheme (Figure 1) and a critical description of the strategies currently adopted to assure and improve CGI quality. Bordogna et al. (2016) and Wiggins et al. (2011) conducted an important overview of quality assessment methods and made significant recommendations for improving CGI quality in research projects. However, these authors only analyzed studies proposing methods for quality assessment of CGI in citizen science projects and did not take into account other CGI sources such as collaborative mapping and social media.

Senaratne et al. (2017) conducted a critical literature review of the existing methods to assess the quality of the main types of CGI: text, image, and map. This review examined methods that are based on theories and discussions in the literature, and provided examples of the practical applicability of all the different approaches. In doing so, the authors provided a general description of the methods used in each paper analyzed; however, they did not create a taxonomy of methods for quality assessment. Moreover, this is a traditional literature review and, as many researchers have pointed out, traditional reviews are prone to bias (i.e., authors may decide only to include studies with which they are familiar or which support their particular standpoint) (Biolchini, Mian, Candida, & Natali, 2005; Mulrow, 1994). In an attempt to minimize this kind of bias, systematic literature reviews (SLRs) have been proposed as a replicable, scientific, and transparent approach to locate the most significant literature about a given topic or discipline (Brereton, Kitchenham, Budgen, Turner, & Khalil, 2007; Kitchenham & Charters, 2007).

Mirbabaie et al. (2016) conducted a systematic literature review on CGI in disaster management. The main goal of this review was to provide information about the quality elements that are used, as well as the methods that are employed to measure these elements. They found that attributes such as “accuracy” and “consistency” are mainly used as criteria for quality assessment, while other factors such as “trustworthiness” are not fully taken into account. However, they did not conduct an in‐depth analysis of the existing methods with regard to their applications and limitations, and were only concerned with the existing methods for disaster management. Moreover, some key databases—such as Web of Science (WoS) and Scopus—were not used by Mirbabaie et al. (2016).

## 4 METHODOLOGY

**Figure 2:**
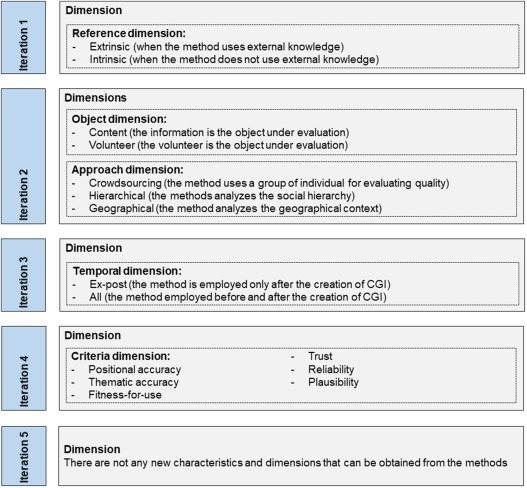
Development process of the taxonomy

For developing the taxonomy, a rigorous and systematic method (Nickerson, Varshney, & Muntermann, 2013) was adopted, providing guidance during the development stage. Since this method is iterative (Figure 2), we selected a subset of the ending conditions (i.e., objective and subjective) that are required to terminate it, as shown in Tables 1 and 2, respectively. We present as follows an overview of our usage of this method and the main stages, while a detailed description of the development process of the taxonomy is provided as Supporting Information.

**Table 1:** Objective ending conditions

Objective ending condition	Comments
All objects or a representative sample of objects have been examined	If all objects have not been examined, then the additional objects need to be studied
At least one object is classified under every characteristic of every dimension	If at least one object is not found under a characteristic, then the taxonomy has a “null” characteristic. We must either identify an object with the characteristic or remove the characteristic from the taxonomy
No new dimensions or characteristics were added in the last iteration	If new dimensions were found, then more characteristics of the dimensions may be identified. If new characteristics were found, then more dimensions may be identified that include these characteristics

*Source*: Adapted from Nickerson et al. (2013).

**Table 2:** Subjective ending conditions

Subjective ending conditions	Questions
Concise	Does the number of dimensions allow the taxonomy to be meaningful without being unwieldy or overwhelming?
Robust	Do the dimensions and characteristics provide for differentiation among objects sufficient to be of interest?
Comprehensive	Can all objects or a (random) sample of objects within the domain of interest be classified?
Extendible	Can a new dimension or a new characteristic of an existing dimension be easily added?
Explanatory	What do the dimensions and characteristics explain about an object?

*Source*: Adapted from Nickerson et al. (2013).

In Iteration 1, we adopted a conceptual‐to‐empirical approach, where the dimensions of the taxonomy are conceptualized without any examination of the objects (Nickerson et al., 2013), but rather based on conceptual distinctions drawn from our literature review of the previous section. As noticed before, some quality assessment methods may use external knowledge for assessing the quality of CGI (i.e., extrinsic), whereas others may not depend on this, but rather require internal knowledge to carry out this task (i.e., intrinsic). We grouped both characteristics into the reference dimension. Since we added one dimension in this iteration, one more iteration was needed.

In Iteration 2, we decided to adopt the empirical‐to‐conceptual approach in which a subset of the objects is classified (Nickerson et al., 2013). On the basis of the studies we found in our SLR (Degrossi, Albuquerque, Rocha, & Zipf, 2017), we identified several quality assessment methods and selected the methods *redundancy of volunteer's contribution* (M2), *volunteer's profile; reputation* (M7), and *spatiotemporal clustering* (M6) based on suitability. On the basis of our understanding of these methods, two new dimensions could be found (i.e., object and approach dimensions). In the object dimension, the object under evaluation could be the information or the volunteer. In the approach dimension, the method analyzes the geographical context (i.e., geographic approach), the social hierarchy (i.e., social approach), or uses a group of people for evaluating quality (i.e., crowdsourcing approach). Since we added new dimensions in this iteration, a further iteration was needed.

In Iteration 3, we adopted the conceptual‐to‐empirical approach once more in order to capture further conceptual distinctions made in the field. As noticed before, quality assessment methods can be distinguished by comparing the time of their application with the time of the creation of the CGI item (Bordogna et al., 2016). Some methods can be employed after a CGI item has been collected (*ex post*), whereas other methods take place before a CGI item has been created (*ex ante*). We grouped both characteristics into the temporal dimension. Since we added one dimension in this iteration, a further iteration was needed.

In Iteration 4, we decided to adopt the empirical‐to‐conceptual approach again because there were still some other methods that needed to be examined. We selected the methods *automatic location checking* (M5), *extraction/learning of characteristics* (M9), *ranking/filtering by linguistic terms* (M10), *volunteer's profile; reputation* (M7), and *scoring volunteered contribution* (M3) from our SLR. On the basis of our understanding of these methods, we identified several quality elements (i.e., positional accuracy, thematic accuracy, fitness‐for‐use, trust, reliability, and plausibility), which were used to measure CGI quality. We grouped them into the criteria dimension. Since one dimension was added in this iteration, one more iteration was needed.

In Iteration 5, we used the empirical‐to‐conceptual approach once again, since there were more methods that needed to be examined. The methods *geographic context* (M1), *expert assessment* (M4), *error detection/correction by crowd* (M8), and *historical data analysis* (M11) were selected from our SLR. After analyzing them, we were unable to identify any new characteristics and dimensions, and thus they were classified in accordance with the characteristics and dimensions outlined above. Since we did not add a new dimension in this iteration and finished examining all the methods from our SLR, it can be concluded that the objective ending conditions were met. Furthermore, the taxonomy met the subjective ending conditions.

The final set of dimensions and characteristics is shown in Table 3. We use them to build the taxonomy of quality assessment methods, which is discussed in the following section.

**Table 3:** Dimensions and characteristics of the taxonomy

Dimension	Characteristics	
Reference	Extrinsic: when the method uses external knowledge Intrinsic: when the method does not use external knowledge	
Object	Content: the information is the object under evaluation Volunteer: the volunteer is the object under evaluation	
Approach	Crowdsourcing: the method uses a group of individuals for evaluating quality Social: the method analyzes the social hierarchy Geographical: the method analyzes the geographical context	
Temporality	Ex‐post: the method is employed only after the creation of CGI All: the method is employed before and after the creation of CGI	
Criteria	Positional accuracy Thematic accuracy Fitness‐for‐use	Trust Reliability Plausibility

## 5 TAXONOMY OF QUALITY ASSESSMENT METHODS

In this section, we introduce the taxonomy resulting from the analysis of the papers selected by our SLR (Degrossi et al., 2017) (Table 4). This taxonomy comprises 11 methods that can be employed for assessing the quality of CGI when authoritative data are not available. Moreover, it consists of a set of dimensions (Table 3) (i.e., reference, object, approach, temporal, and criteria dimensions) that represent an abstraction of the main attributes of each method. Each dimension has a set of possible values, which are called the characteristics of a method. The dimension “approach,” for instance, comprises three possible characteristics (i.e., crowdsourcing, social, and geographical approaches). Each method was classified in accordance with these dimensions and characteristics (Table 5).

**Table 4:** Summary of the quality assessment methods of the taxonomy and corresponding primary studies

ID	Method	Description	Primary Studies
**M1**	Geographic context	Investigating the area surrounding the location of CGI to determine its geographical features and employ them to assess the quality of CGI.	Senaratne et al. (2013); Zielstra & Hochmair (2013)
**M2**	Redundancy of volunteers' contribution	Requesting several volunteers to provide information about the same geographic feature to find out if or not there is a convergence of the information produced by different volunteers.	Comber et al. (2013); Foody (2014); See et al. (2013)
**M3**	Scoring volunteered contribution	Asking volunteers to rate every piece of CGI that is contributed by other volunteers.	Lertnattee et al. (2010)
**M4**	Expert assessment	Submitting CGI to experts who are responsible for checking the information content and correcting it if necessary.	Foody et al. (2013); Karam & Melchiori (2013)
**M5**	Automatic location checking	Estimating the quality of CGI by the distance between geocoded coordinates, obtained from multiple geocoding services, and the location (i.e., an address) provided by the volunteer.	Cui (2013)
**M6**	Spatiotemporal clustering	Creating spatiotemporal clusters of CGI elements using prior information about a phenomenon of interest and, later, evaluating the significance of the resulting clusters for a specific purpose.	Longueville et al. (2010)
**M7**	Volunteer's profile; reputation	Analyzing volunteer's profile or reputation and using it to estimate the quality of CGI.	Bishr & Janowicz (2010); Bishr & Kuhn (2013); Bodnar et al. (2014)
**M8**	Error detection /correct by crowd	Several volunteers acting as gatekeepers and, thus, correcting errors introduced by other volunteers.	Haklay et al. (2010)
**M9**	Extracting/learning of characteristics	Extracting characteristics from each type of geographic feature, learning the information implicit in them and, later, using the information to estimate the quality of CGI.	Ali & Schmid (2014); Jilani & Corcoran (2014); Mohammadi & Malek (2015)
**M10**	Ranking/filtering by linguistic terms	Evaluate CGI items based on different criteria that are expressed linguistically, rank them in degrees of criteria satisfaction and, later, filter them based on the constraints of the application domain.	Bordogna et al. (2014)
**M11**	Historical data analysis	Deriving (intrinsic) indicators from the history of the data and, later, using them to make statements regarding the quality of CGI.	Keßler & de Groot (2013)

**Table 5:** Taxonomy of quality assessment methods

		Object		Approach			Reference		Temporality		Criteria					
ID	Method	INF	VOL	GEO	CWD	SOC	EXT	INT	EP	ALL	Positional accuracy	Thematic accuracy	Fitness for use	Trust	Reliability	Plausibility
M1	Geographic context	x		x			x		x		x					
M2	Redundancy of volunteers’ contribution	x			x		x		x			x				
M3	Scoring volunteered contribution	x			x		x		x						x	
M4	Ranking volunteers’ contribution	x			x		x		x			x				
M5	Automatic location checking	x		x			x		x		x					
M6	Spatiotemporal clustering	x		x				x	x						x	
M7	Volunteer's profile; reputation		x			x		x		x				x		
M8	Error detection/correct by crowd	x			x		x		x		x					
M9	Extracting/learning of characteristics	x		x				x		x		x				
M10	Ranking/filtering by linguistic terms	x		x				x		x			x			
M11	Historical data analysis	x			x			x	x					x		

INF = information; VOL = volunteer; GEO = geographic approach; CWD = crowdsourcing approach; SOC = social approach; EX = extrinsic; INT = intrinsic; EP = ex‐post.

In the following sections, we present a more detailed description of the existing methods to assess CGI quality. We also provide practical examples of their applicability and discuss any drawbacks that might prevent their employment.

### 5.1 Geographic context

###### Description

Each place has its own distinguishing characteristics. The basic idea of the “geographic context” method consists of investigating the area surrounding the location of CGI to determine its geographical features and employ them for assessing the likelihood that the citizen‐generated data is consistent with those features. Both physical and human geographical features (e.g., hydrological, geomorphological, socio‐economical, demographic, etc.) could potentially be used in this method.

###### Example

Senaratne et al. (2013) examined the geographic context around Flickr photographs to determine which areas can be viewed from the CGI location, or more specifically, whether the point of interest (POI) lies within the line of sight departing from the coordinates given in the CGI geotag. Similarly, Zielstra and Hochmair (2013) carried out an investigation to evaluate the positional accuracy of geotagged photos from Flickr and Panoramio. Here, the positional accuracy was estimated by measuring the distance between the image position and the estimated camera position based on the imagery metadata.

###### Limitation

A key factor when applying this method is determining which geographical features are related to the CGI content, since they must be matched. Furthermore, establishing reliable geographic relations is not a trivial problem, since they may vary in accordance with the physical area and domain. Finally, this method may also be constrained by the unsatisfactory quality of the reference data (e.g., the background map) used for the measurements.

### 5.2 Redundancy of volunteers’ contribution

###### Description

This method involves requesting several volunteers to provide information about the same geographic feature independently. Later, CGI quality is determined by comparing the volunteers’ contributions in order to find out whether or not there is a convergence of the information independently produced by different volunteers. In other words, the CGI quality is assumed to be higher when there is agreement among volunteers with regard to the information content.

###### Example

Comber et al. (2013) estimated the degree of reliability of CGI by asking volunteers (i.e., experts, postgraduate students, scientists, and novices) to identify the type of land cover based on satellite imagery of a series of locations. Similarly, See et al. (2013) employed this method to evaluate the accuracy and consistency of volunteers when labeling land cover and determining the human impact on the environment. In contrast, Foody (2014) explored the redundancy of contributions to situations in which a large proportion of data is provided by poor sources and/or is incomplete.

###### Limitation

This type of analysis is applicable to situations where it is practical to obtain several versions of the volunteered information independently (i.e., in which different volunteers provide information about the same geographic feature(s) independently and the quality can be associated with the degree of agreement among them). However, it is worth noting that the ability to reach an agreement among volunteers will also depend on the difficulty of the task(s). In other words, there is a greater chance of reaching an agreement when the tasks are “easy” than when they are moderately hard to difficult (Albuquerque et al., 2016; Salk, Sturn, See, & Fritz, 2017). Moreover, since the information is based on the knowledge of volunteers, it could cause problems as some of it may be less reliable and of poorer quality (Comber et al., 2013).

### 5.3 Scoring volunteered contribution

###### Description

In this method, volunteers are asked to rate every piece of CGI that is contributed by other volunteers. A score is thus calculated from the individual rates given and assumed to represent “content quality.”

###### Example

Lertnattee, Chomya, and Sornlertlamvanich (2010) used a score calculated from the number of clicks on the button “Vote” given by members of the community to herbal information, a type of information related to medicinal herbs.

###### Limitation

The success of the method depends on how successful a platform is in gathering rates from volunteers, since it might require a large number of raters to arrive at a score that truly represents the “content quality.”

### 5.4 Expert assessment

###### Description

Feedback and contributions from individuals that are familiar with an area or domain may greatly assist in estimating the quality of CGI (Karam & Melchiori, 2013). In this method, CGI is submitted to experts (i.e., individuals who have a greater knowledge of the geographic area or domain and are responsible for checking the information content and correcting it if necessary). Later, all the CGI are ranked on the basis of the corrections made by the individuals. When CGI is ranked in a lower position, it indicates the presence of malicious and/or wrong corrections. In other words, it is CGI of a poorer quality.

###### Example

Karam and Melchiori (2013) ranked CGI by employing four metrics: (a) the historical records of activities carried out by a volunteer; (b) the number of activities carried out by experts; (c) the feedback received after the change was made; and (d) the score of the user that submitted the information.

###### Limitation

A drawback of this method is that it requires (expert) individuals who are able to check the CGI provided by volunteers.

### 5.5 Automatic location checking

###### Description

The location of a volunteer can be ascertained in different ways, such as GPS (global positioning system), manual georeferencing, or an address. However, the last of these could contain errors or typos. A way of automatically determining the correct location of a volunteer is to compare geocoded coordinates, from multiple geocoding services, with each other. Hence, the address should be submitted to several geocoding services, which will result in coordinates that are represented by latitude and longitude. To qualify as reference data, the different geocoding services should yield concordant results within a certain distance (Cui, 2013). The quality is, thus, estimated as the distance between the geocoded coordinate and the location provided by the volunteer.

###### Example

Cui (2013) employed automatic location checking to determine the spatial accuracy of the location of farmers’ markets.

###### Limitation

The reference data and geocoded methods used in the geocoding service might contain errors and, thus, lead to erroneous results (Cui, 2013). Hence, the use of multiple geocoding vendors’ services can improve the reliability of the resulting geocoded coordinates. Furthermore, the CGI location must be provided through an address or place name that will be submitted to the geocoding services.

### 5.6 Spatiotemporal clustering

###### Description

CGI quality can be addressed by aggregating information from several volunteers (Mummidi & Krumm, 2008) and, later, by evaluating the significance of the resulting clusters for a specific purpose (Longueville, Luraschi, Smits, Peedell, & Groeve, 2010). Thus, instead of checking the quality of a single CGI element, all elements are evaluated as a whole (i.e., the quality of the CGI cluster is assessed).

This method consists of creating spatiotemporal clusters of CGI elements using prior information about a phenomenon of interest. The clusters are created on the basis of the assumption that “CGI elements created at the same place and time refer to the same event” (Longueville et al., 2010).

The process starts by creating temporal clusters, which are the CGI elements clustered in several temporal classes. After this, the temporal classes are divided into sub‐classes in compliance with spatial criteria. These steps convert raw CGI of unknown quality into spatiotemporal clusters, the importance of which can be quantified by means of a ranking score, which reflects the likelihood that an event took place in the time period and area that each cluster refers to.

###### Example

Longueville et al. (2010) employed spatiotemporal clustering to assess the likelihood that a flood event took place (i.e., to locate inundation areas of flood events that occurred in the United Kingdom between January 1st, 2007 and March 31st, 2009).

###### Limitation

As Longueville et al. (2010) point out, when employing this method, a large dataset must be made available, since a low amount of data prevents a robust statistical analysis from being conducted. Hence, this method is less suitable for sparsely populated areas (e.g., rural areas), where only a few VGI elements are available.

### 5.7 Volunteer's profile; reputation

###### Description

The volunteer is an important factor in the quality of CGI, since his/her knowledge and background can have an influence on the data produced. By employing this method, the volunteer's profile or reputation can be analyzed and used as a basis to estimate the quality of CGI. Bishr and Janowicz (2010) argue that if the volunteer has a reputation for being trustworthy, this means that his or her contribution is likely to be trustworthy too.

###### Example

Bodnar, Tucker, Hopkinson, and Bilen (2014) analyzed volunteers’ profiles to establish the veracity of volunteered contributions with regard to four security‐related events that took place in the U.S. In contrast, Bishr and Kuhn (2013) employed this method to assess the trustworthiness of volunteers’ statements regarding the quality of water from a well.

###### Limitation

This method can be adversely affected if some of the metadata is missing or inaccurate (i.e., the metadata concerning a volunteer's profile). Thus, before employing it, it is essential to check if reliable information about the volunteers is available. Another limitation is that this method does not consider the extent to which users have the necessary skills for a particular task or context. Volunteers may be reliable at producing data about the surroundings in which they live, but when generating data about geographic areas for which they do not have any contextual knowledge, they may produce less reliable geographic information (Klonner, Eckle, Usón, & Höfle, 2017).

### 5.8 Error detection/correct by crowd

###### Description

This method is based on the so‐called Linus's law, according to which “given enough eyeballs, all bugs are shallow” (Raymond, 1999). In the case of CGI, this can be understood as “given enough volunteers, all errors can be identified and corrected.”

The basic idea behind this method is that a single individual might unintentionally introduce an error in a crowdsourcing‐based platform. Later, other people might notice this error and correct it, and hence the community of volunteers acts as gatekeepers. This method is different from the redundancy of volunteers’ contribution (Section 5.2), since it is not based on an aggregation of information that is independently provided by volunteers, but rather relies on peer verification of the information produced by other volunteers as a self‐correcting mechanism.

###### Example

Haklay, Basiouka, Antoniou, and Ather (2010) investigated whether Linus's law applies to the positional accuracy of OSM data (i.e., if the positional accuracy of a given geographic feature in OSM increases incrementally with the number of volunteers).

###### Limitation

As Linus's law states, there should be enough eyeballs (volunteers) to identify and correct the errors. However, this could be a drawback, since a large number of people may be needed to achieve good quality. This can be especially problematic in sparsely populated areas, such as rural areas where the number of volunteers is small, as well as in virtual communities that have a small number of users.

### 5.9 Extraction/learning of characteristics

###### Description

Each geographic feature has characteristics (i.e., shape, size, etc.) which can be used as a classification criterion. This method consists of extracting these characteristics from each type of geographic feature, learning the information implicit in them, and, later, using the information to estimate the quality of CGI. For instance, distinct characteristics can be extracted and learned from CGI with corresponding reference data and, later, used to estimate the quality of CGI with no corresponding reference data.

###### Example

Ali and Schmid (2014) designed a classifier that learns the correct class of existing entities (i.e., parks and gardens) on the basis of their characteristics (e.g., size) and used it to predict the correct class of a new entity. Similarly, Jilani and Corcoran (2014) extracted geometrical and topological properties of OSM street network data that are representative of their semantic class, to infer the “road class” from the new data. Finally, Mohammadi and Malek (2015) estimated the positional accuracy of OSM data without corresponding reference data by extracting patterns from OSM data that have corresponding reference data.

###### Limitation

This method does not depend on any external source when being employed. However, a large amount of data is required to properly learn the characteristics of geographic features. Moreover, it is context‐dependent, since geographic features in the same region might have more similar characteristics to those in different regions.

### 5.10 Ranking/filtering by linguistic terms

###### Description

The underlying principle of this method is the need to express the quality criteria linguistically. The linguistic terms are used to specify the desired values of the quality indicators and, together, these comprise a schema for quality evaluation. Each CGI item is first evaluated on the basis of each criterion that is expressed linguistically and, later, ranked in degrees of criteria satisfaction. Finally, CGI items are filtered by being subject to the constraints of the application domain.

###### Example

Bordogna, Carrara, Criscuolo, Pepe, and Rampini (2014) employed this method to filter CGI items (i.e., pictures) for a citizen science project on glaciology.

###### Limitation

One critical factor in this method is the schema for quality evaluation, since this is changed by each intended use of the CGI items. In other words, the schema depends on the application domain.

### 5.11 Historical data analysis

###### Description

In special cases, CGI comes with historical data. In OSM, for instance, a new version of an object is created whenever its geometry is changed. From the history of the data, it is possible to derive (intrinsic) indicators that allow approximate statements to be made regarding data quality (Barron et al., 2014). An example of an indicator is the number of contributors in a given area, since it has been demonstrated that this has an influence on the quality (Haklay et al., 2010). Moreover, the historical data can be analyzed to identify patterns and make predictions of quality elements.

###### Example

Keßler and de Groot (2013) produced a set of indicators based on historical data (i.e., number of versions, contributors, confirmations, tag corrections, and rollbacks) to assess the quality of OSM data in Muenster, Germany. Positive indicators (e.g., a high version number) were shown to correlate with high‐quality CGI.

###### Limitation

This is an alternative method when no ground‐truth data is available. However, a certain amount of historical data must be available before it can be applied. Otherwise, the value of resulting statements may be limited.

## 6 DISCUSSION

This article presents a taxonomy of quality assessment methods for CGI. In contrast with existing works, as shown in Section 3, this taxonomy summarizes what methods can be employed to assess the quality of CGI when authoritative data is not available. In creating this taxonomy, we took into account different types of CGI source (i.e., social media, crowd sensing, and collaborative mapping). As a result, the taxonomy can be useful for quality assessment in a larger number of crowdsourcing‐based platforms. Thus, this article can be regarded as an extension of previous work (Bordogna et al., 2016; Wiggins et al., 2011), which only includes methods for assessing the quality of CGI in citizen science projects (i.e., crowd sensing).

Some of the methods presented in the taxonomy have already been identified in previous studies (Bordogna et al., 2016; Mirbabaie et al., 2016; Senaratne et al., 2017; Wiggins et al., 2011), such as *redundancy of volunteers’ contribution*, *scoring volunteered contribution*, *expert assessment*, *volunteer's reputation*, *ranking/filtering by linguistic terms*, and *historical data analysis*. Other methods have not been identified as such in the literature so far (i.e., a contribution of the present work is to identify a set of new methods to assess the quality of different types of CGI when there is no authoritative data).

As well as in previous studies, we briefly described how each method works, classified them according to their temporality and the approach employed, and presented practical examples. In addition, we classified them in accordance with the type of reference dataset and the object under evaluation, and discussed their limitation(s), which may prevent their employment. Moreover, we analyzed the CGI sources in which each method could be employed. With the exception of four methods (i.e., *scoring volunteered contribution*, *spatiotemporal clustering*, *error detection/correct by crowd*, and *historical data analysis*), the majority of the methods can be employed with all CGI sources (i.e., social media, crowd sensing, and collaborative mapping).

The method *scoring volunteered contribution* (M3) can be employed for some crowd sensing platforms, since it is possible to measure a score and attach it to the information. On the other hand, in some collaborative mapping and social media platforms, it is not possible to directly measure a score but only to give indirect indicators of information quality; this is owing to the idiosyncrasies of these platforms.

The method *spatiotemporal clustering* (M6) can be employed for crowd sensing and social media platforms. In these platforms, volunteers are able to share information about a specific event. Moreover, this set of information is usually created in the same area and during a certain period of time. In contrast, in collaborative mapping platforms, the volunteers are not able to share information about an event, since these platforms usually collect data about less dynamic geographic features, such as roads and buildings.

The method *error detection/correct by crowd* (M8) cannot be employed for social media platforms since no one is allowed to correct the contributions of anyone else. However, it can be applied to some collaborative mapping and crowd sensing platforms if permission is granted to volunteers to edit the information.

Finally, the method *historical data analysis* (M11) can only be employed in crowdsourcing platforms that keep records of all the changes of a geographic feature, such as some collaborative mapping and crowd sensing platforms, owing to its main characteristic (i.e., the use of historical data). Social media platforms, however, do not keep any records, and this prevents them from using this method.

One advantage of our taxonomy is the fact that it is domain‐independent (i.e., it can be employed in any field of study). This is different from the work of Mirbabaie et al. (2016), which is focused exclusively on the application domain of disaster management. Another advantage is the systematic process employed to develop our taxonomy. As a result of this, we were able to identify dimensions and characteristics that have not been identified in previous studies (Bordogna et al., 2016; Senaratne et al., 2017), such as object, reference, and criteria dimensions. Finally, our taxonomy provides a synthesis of the existing methods being employed to assess CGI quality, which adds a systematization of research in this area and thus complements previous works which have focused on summarizing existing studies in the literature related to quality assessment of CGI (Mirbabaie et al., 2016; Senaratne et al., 2017; Wiggins et al., 2011).

An analysis of the methods presented in the taxonomy reveals opportunities for the development of new indicators and other methods to assess the quality of CGI. Furthermore, it allows researchers working with a particular type of CGI (e.g., social media) to learn about methods developed and employed in different types of CGI and application domains that could be transferred to their research focus.

As pointed out in previous sections, quality assessment relies on the type and amount of data, the application domain, and the reference dataset available. As a consequence, only a few methods can be employed in different CGI sources and application domains. However, before their employment, there is a need to evaluate their appropriateness. If the methods available are not suitable for a specific purpose, then future investigation should be carried out in order to develop new method(s).

Unlike in existing works, our taxonomy was developed in a systematic way (i.e., the identification of existing methods through an SLR and development of the taxonomy). However, it still presents some limitations. SLR is a rigorous and systematic methodology, but there are some threats to its validity. These have been minimized by selecting several synonyms for both the main keywords, with the aim of discovering all the primary studies in the area. However, we did not employ all synonyms in all electronic databases owing to their idiosyncrasies; in other words, we had to exclude some synonyms in some search engines because we could not identify relevant studies with them. The limited number of selected studies might be seen as a consequence. In addition, the number of studies included might have been affected by language restrictions, as only studies written in English and Portuguese were taken into account. Thus, it is possible that some relevant studies were not included in this work.

## 7 CONCLUSIONS

In this article, we propose a taxonomy of methods for assessing the quality of CGI in the absence of authoritative data. This first involved looking at the state‐of‐the‐art by conducting a systematic literature review, and several works were found that employ quality assessment methods for CGI. After this, we investigated each method to determine its characteristics and, thus, be in a position to create our taxonomy. Following this, we described each method, discussing its limitation(s) and potential application.

Our taxonomy is aimed at assisting quality assessment in new and existing crowdsourcing‐based platforms. Assessment is an important stage in all CGI, since the information comes from unknown sources and is of unknown quality. As well as this, the scientific community can also benefit from the results of our taxonomy, because it provides an overview of existing methods, but also offers scope for future research projects.

## Supporting information

Additional Supporting Information may be found online in the supporting information tab for this article.

Supporting Information
