# Supplementary material for: A taxonomy of quality assessment methods for volunteered and crowdsourced geographic information
Source: Trans GIS. 2018 Apr 19;22(2):542–60. doi: 10.1111/tgis.12329 (PMC5993263; doi:10.1111/tgis.12329)
Supplement: Supplementary file 1 — Supporting Information [file TGIS-22-542-s001.docx]

**Appendix A Algorithms in existing map generalization software**

According to Nickerson, Varshney, & Muntermann (2013), a taxonomy is a set of *n* dimensions each consisting of mutually exclusive and collectively exhaustive characteristics. The mutually exclusive and collectively exhaustive restrictions mean that no object can have two different characteristics in a dimension and each object must have one of the characteristic in a dimension. A well-conceived method was adopted for the development of the taxonomy that was devised by Nickerson, Varshney, & Muntermann (2013) and provides guidance for researchers during the development stage.

The definition of the characteristics of the taxonomy is based on a meta-characteristic (i.e. the most comprehensive characteristic), which serves as the basis for choosing them. In particular, the meta-characteristic is defined in terms of the purpose of the taxonomy, which, in turn, depends on its expected use. This can be carried out by determining who the users are and what use they could make of the taxonomy. The users of our taxonomy are both researchers and designers of crowdsourcing-based platforms and are interested in the quality assessment methods that can be employed on those platforms when no authoritative data is available. Thus, the purpose of our taxonomy is to distinguish between quality assessment methods on the basis of their ability to assess the quality of CGI. With the aid of this taxonomy, we seek to assist the users in understanding the unique features of the current methods and suggest ways of devising new methods. Thus, the meta-characteristic for designing this taxonomy is the way in which the method assesses the quality of CGI.

In order to determine the dimensions and characteristics of the taxonomy, we first defined a set of objective and subjective conditions that are necessary to terminate the method since it is iterative. The main ending conditions that the taxonomy must satisfy are mutually exclusive and collective exhaustive characteristics. Additionally, our taxonomy must also satisfy three objective conditions (Table 1) and five subjective ending conditions (Table 2) which represent the qualitative attributes of a useful taxonomy.

| **Objective ending condition** | **Comments** |
| --- | --- |
| All objects or a representative sample of objects have been examined | If all objects have not been examined, then the additional objects need to be studied |
| At least one object is classified under every characteristic of every dimension | If at least one object is not found under a characteristic, then the taxonomy has a ‘null’ characteristic. We must either identify an object with the characteristic or remove the characteristic from the taxonomy |
| No new dimensions or characteristics were added in the last iteration | If new dimensions were found, then more characteristics of the dimensions may be identified. If new characteristics were found, then more dimensions may be identified that include these characteristics |

Table 1: Objective-ending conditions (Adapted from: Nickerson et al. (2013)).

After the definition of the meta-characteristic and the ending conditions, the identification of the dimensions and characteristics can begin. This entails adopting two approaches (i.e., empirical and conceptual approaches) in the iterations so as to have several perspectives. In the conceptual-to-empirical approach, the dimensions of the taxonomy are conceptualized without examining the objects (Nickerson et al., 2013). On the other hand, in the empirical-to-conceptual approach, a subset of the objects are classified (Nickerson et al., 2013). In the following, we describe the iterations carried out for the development of the proposed taxonomy, as also shown in Fig. 1.

| **Subjective ending conditions** | **Questions** |
| --- | --- |
| Concise | Does the number of dimensions allow the taxonomy to be meaningful without being unwieldy or overwhelming? |
| Robust | Do the dimensions and characteristics provide for differentiation among objects sufficient to be of interest? |
| Comprehensive | Can all objects or a (random) sample of objects within the domain of interest be classified? |
| Extendible | Can a new dimension or a new characteristic of an existing dimension be easily added? |
| Explanatory | What do the dimensions and characteristic explain about an object? |

Table 2: Subjective ending conditions (Adapted from: Nickerson et al. (2013)).

In Iteration 1, we adopted a conceptual-to-empirical approach where the dimensions of the taxonomy are conceptualized without any examination the objects (Nickerson et al., 2013), but rather based on conceptual distinctions draw from our literature review of the previous section. As noticed before, some quality assessment methods may use external knowledge for assessing the quality of CGI (i.e., extrinsic), whereas others may not depend on this, but rather require internal knowledge to carry out this task (i.e., intrinsic). We grouped both characteristics into the reference dimension. Since we added one dimension in this iteration, one more iteration was needed.

In Iteration 2, we decided to adopt the empirical-to-conceptual approach in which a subset of the objects is classified (Nickerson et al., 2013). On the basis of the studies we found in our SLR (Degrossi, Albuquerque, Rocha, & Zipf, 2017), we identified several quality assessment methods and selected the methods redundancy of volunteer’s contribution (M2), volunteer’s profile; reputation (M7), and spatiotemporal clustering (M6) based on suitability. On the basis of our understanding of these methods, two new dimensions could be found, i.e., object and approach dimensions. In the object dimension, the object under evaluation could be the information or the volunteer. In the approach dimension, the method analyzes the geographical context (i.e., geographic approach), the social hierarchy (i.e., social approach) or uses a group of people for evaluating quality (i.e., crowdsourcing approach). Since we added new dimensions in this iteration, a further iteration was needed.

In Iteration 3, we adopted the conceptual-to-empirical approach once more in order to capture further conceptual distinctions made in the field. As noticed before, quality assessment methods can be distinguished by comparing the time of their application to the time of the creation of the CGI item (Bordogna et al., 2016). Some methods can be employed after a CGI item has been collected (ex-post), whereas other methods take place before a CGI item has been created (ex-ante). We grouped both characteristics into the temporal dimension. Since we added one dimension in this iteration, a further iteration was needed.

In Iteration 4, we decided to adopt the empirical-to-conceptual approach again because there were still some other methods that needed to be examined. We selected the methods automatic location checking (M5), extraction/learning of characteristics (M9), ranking/filtering by linguistic terms (M10), volunteer’s profile; reputation (M7) and scoring volunteered contribution (M3) from our SLR. On the basis of our understanding of these methods, we identified several quality elements (i.e., positional accuracy, thematic accuracy, fitness-for-use, trust, reliability, and plausibility), which were used to measure CGI quality. We grouped them into the criteria dimension. Since one dimension was added in this iteration, one more iteration was needed.

In Iteration 5, we used the empirical-to-conceptual approach once again since there were more methods that needed to be examined. The methods geographic context (M1), expert assessment (M4), error detection/correction by crowd (M8), historical data analysis (M11) were selected from our SLR. After analyzing them, we were unable to identify any new characteristics and dimensions and thus they were classified in accordance with the characteristics and dimensions outlined above. Since we did not add a new dimension in this iteration and finished examining all the methods from our SLR, it can be concluded that the objective ending conditions were met. Furthermore, the taxonomy met the subjective ending conditions.


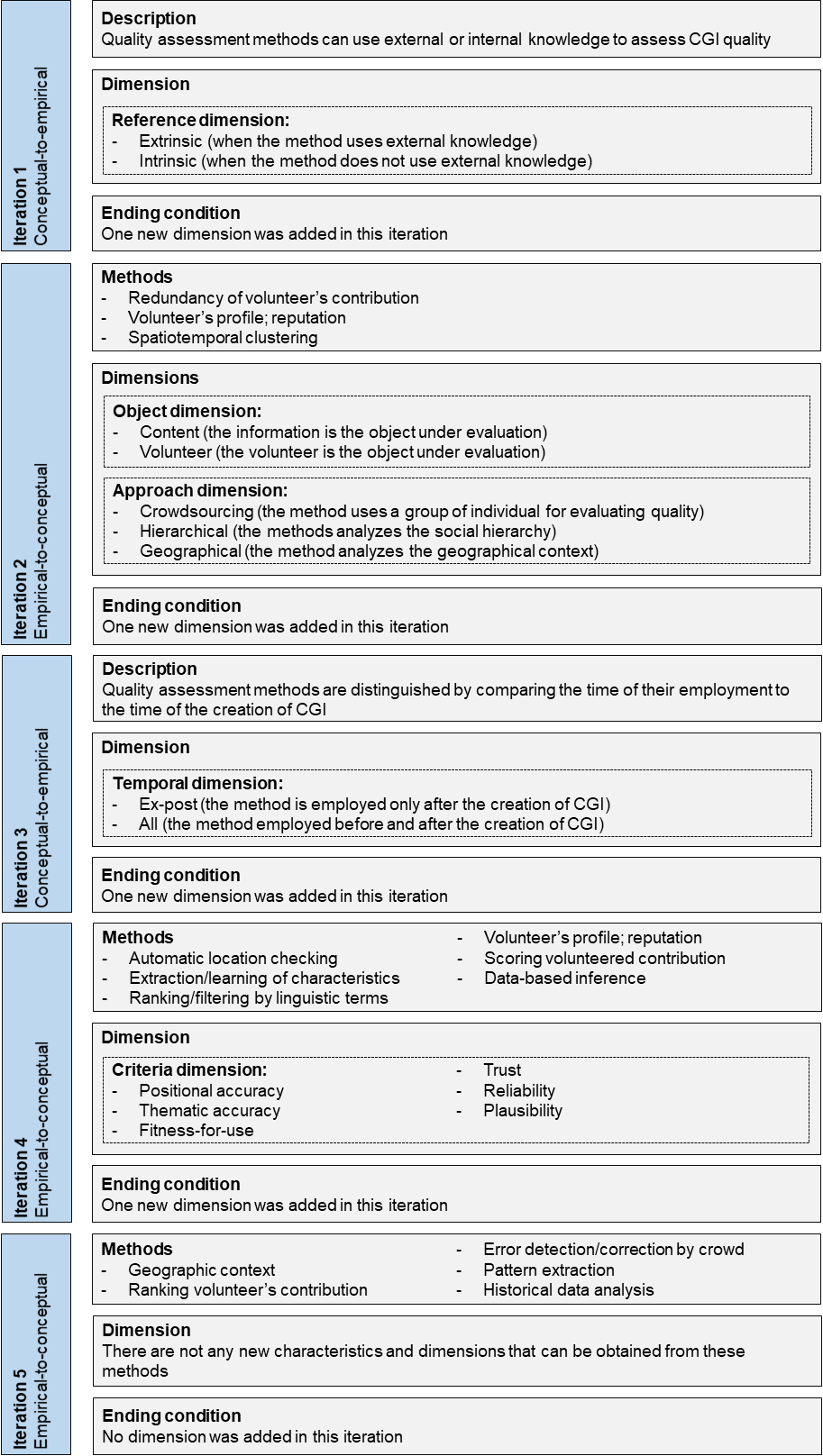


Fig.1: Complete development process of the taxonomy
